# Energy poverty and its relationship with health: empirical evidence on the dynamics of energy poverty and poor health in Australia

**DOI:** 10.1007/s43546-021-00149-3

**Published:** 2021-09-29

**Authors:** Heather Brown, Esperanza Vera-Toscano

**Affiliations:** 1 Newcastle University, Population Health Sciences Institute, Royal Victoria Infirmary, Ridley 1, Newcastle Upon Tyne, NE1 4LP UK; 2 University of Melbourne, Level 4, FBE Building, 111 Barry Street, Carlton, VIC 3010 Australia

**Keywords:** Energy poverty, Health, Dynamic bivariate probit, Australia, D31, D63, I14

## Abstract

Does poor health increase the likelihood of energy poverty or vice versa creating a vicious poverty trap? We use data from the Household, Income and Labour Dynamics of Australia (HILDA) survey from 2005–2018 to explore if these two processes are dynamically related across a number of subjective and objective measures of physical and mental health as well as subjective and objective measures of energy poverty. We employ univariate dynamic models, introduce controls for initial conditions, and explore inter-dependence between energy poverty and health using a dynamic bivariate probit model. Our results show that controlling for initial conditions impacts on the magnitude and significance of the lagged coefficients. We only find cross-dependency effects between energy poverty and health for subjective measures of energy poverty. This suggests that individuals’ feelings about being in energy poverty may impact on their health leading to poor health/energy poverty traps. Targeting individuals in financial stress/debt may be one way to reduce these poor health/energy poverty traps.

## Introduction

The inability to access energy is a global issue that currently affects approximately 30% of the world’s population (Halff et al. 2014). There is no universally accepted definition of energy poverty. But, across higher income countries, most measures encompass a situation in which a person cannot obtain the necessary energy to keep their home at a comfortable temperature and meet their basic needs because of inadequate resources or living conditions which is measured either subjectively or objectively.^1^ The prevalence is higher in cooler European countries with a poor housing stock such as the UK where approximately 10.3% of English households are in energy poverty.^2^ However, the problem is not limited to colder countries as fuel poverty^3^ can also refer to a situation where individuals cannot afford to cool their home or have a properly cooked meal. In warmer countries, such as Australia using a similar definition of energy poverty as in the UK, the prevalence is approximately 2.5%.^4^

There is an increasing body of empirical evidence from an economic perspective showing a relationship between energy poverty and poor general and mental health (Churchill and Smyth 2020; Llorca et al. 2020; Kahouli 2020; Rodríguez-Álvarez et al. 2019; Thomson et al. 2017; Grey et al. 2017; Lacroix and Chaton 2015). In the UK, inability to adequately heat one’s home is associated with poor mental health and physical health in both adolescents and adults (Thomson et al. 2017; Marmot 2010). Similar associations between energy poverty and health have been found in other European countries such as Spain (Llorca et al. 2020), France (Kahouli 2020; Lacroix and Chaton 2015), across the European Union and in particular Mediterranean and Eastern European countries (Oliveras et al. 2020). Churchill and Smyth (2020) find a significant relationship between health and energy poverty for Australia.

Much of the literature relies on cross-sectional data (Oliveras et al. 2020; Thomson et al. 2017; Lacroix and Chaton 2015); thus, these studies can only estimate associations of energy poverty and health but not establish a causal relationship. More recent studies in the field such as Churchill and Smyth (2020) and Kahouli (2020) attempt to estimate a causal relationship of energy poverty using longitudinal panel data to address the bias of energy poverty being endogenous. Both of these studies focus on endogeneity bias stemming from omitted variable bias rather than reverse causality. Churchill and Smyth (2020) employ an instrument variable (IV) approach in which they use energy prices as an instrument. Household energy prices have risen significantly across Australia since 2007 by 76% for electricity and 53% for gas (Department of Industry, Innovation and Science 2016; Australian Competition and Consumer Commission 2018). They assume that energy prices only impact on energy poverty but not health. Churchill and Smyth (2020) acknowledge that rising energy prices will influence the household budget, thus, potentially impacting on both energy and health expenditure. They also note that energy prices may reflect economic performance of the economy and thus, mental health related to future financial security. They attempt to remove this contingency from the unobserved error term, thus, violating the IV assumptions, by including gross state domestic product as a control variable. They find for Australia that a standard deviation increase in energy poverty is associated with a decline in health of between 0.10 and 0.30 standard deviation. Kahouli (2020) employs a fixed effects IV approach using energy prices and if the individual received a housing refit subsidy. He assumes that any of the impact of a housing refit subsidy, which he states in France are mostly spent on insulation, will only have an indirect effect on health. However, evidence from a randomized control study (Howden-Chapman et al. 2007) had found direct effect of insulation on health, making this assumption debatable. Notwithstanding, Kahouli (2020) found depending upon how energy poverty was measured it led to between a 10 and 13% decrease in self-reported health.

We argue that estimating a causal relationship between energy poverty to poor health is an important contribution to the literature; but that this is likely to be an over-simplification, as there is likely to be an inter-related dynamic relationship between energy poverty and health which has not been adequately accounted for or described in the literature. In other words, it is important to not only consider omitted variable bias but reverse causality ant the inter-dependence of energy poverty and poor health as well. Poverty and the determinants of poverty are complex and estimating only part of that relationship may lead to inefficient poverty reduction strategies. There is evidence showing an interdependent relationship with different dimensions of poverty. For example, Devicienti and Poggi (2011) explore the inter-related dynamic process of poverty and social exclusion. Ribar and Hamrick (2003) explore the dynamics of poverty and food insecurity. Sweet et al. (2013) and Clayton et al. (2015) demonstrate a dynamic and interdependent relationship between debt and health.

Poor health may cause energy poverty and energy poverty may cause poor health. People with certain health conditions such as cardiovascular, pulmonary, or respiratory diseases, and arthritis will be more sensitive to temperature extremes. If they are suffering from energy poverty and have a house that is too cold or too hot this may exacerbate their symptoms (World Health Organisation 2017; Bull et al. 2010; Liddell and Morris 2010). People with poor health may also need to spend more time at home increasing their demand for energy or their health condition may lead to increasing demands for electricity such as a dialysis machine (Büchs et al. 2018). If people have a greater demand for electricity this will lead to higher costs increasing the likelihood of these households suffering from energy poverty. The social determinants of health-non-medical factors such as the conditions that people are born, grow, live, work, and age such as income, education, employment, and housing. These factors explain approximately 30–55% of health outcomes (World Health Organisation 2021). People who live in poor housing that is energy inefficient, with instable and low-quality employment are more likely to suffer from poor health. These factors which contribute to poor health also contribute to increased risk for energy poverty. Thus, social determinants of health are an important predictor and determinant of energy poverty (Jessel et al. 2019). Household size and composition will interact with the social determinants of health and be key determinants of energy poverty (Großmann and Kahlheber 2017).

There are several determinants of energy poverty which are influenced by and related to the social determinants of health. People who live in low income and ethnic minority neighbourhoods are at increased risk for exposure to environmental hazards, face a lack of investment in housing stock and poorer initial quality of housing stock (Jessel et al. 2019). In many cases these neighbourhoods are also subject to other aspects of deprivation such as reduced opportunities for education, transport, and employment which all contribute to increased risk of energy poverty (Marmot et al. 2005). Thus, the causes of energy poverty and poor health are complementary and can be seen as mutually reinforcing.

There is some empirical evidence supporting this idea of poor health and energy poverty traps. In Australia, 60.4% of households who cannot afford to adequately heat their home include at least one person with a long-term health condition or disability (Azpitarte et al. 2015). Approximately half (48.3%) of all household who cannot afford to heat their home over the long term have at least one occupant with physical limitations limiting their capacity to work. Importantly, a large majority (73.8%) of people with poor mental health are also in persistent energy payment difficulty (Victorian Council of Social Service 2018). This provides further empirical support that energy poverty is associated with poor health and poor health is associated with higher risk of being in energy poverty. Given the global climate emergency, it is important to better understand the observed correlation between health and energy poverty. There may be an economic, environmental, and health benefit of improving housing stock and energy efficiency making a clear economic case for these policies which could be lost by focusing solely on the impact of energy poverty on health.

The aim of this paper is to explore the dynamics and persistence of energy poverty and health. We model the two processes jointly in a discrete sequential equation model where we assume that the health risk affects the energy poverty risk and vice versa. We estimate a dynamic bivariate probit model in which we explicitly account for the joint distribution of unobserved heterogeneity and we control for the initial conditions as in Wooldridge (2005). Similar to Alessie et al. (2004) we assume sequential causality, in which the last period’s health is assumed to affect the current period’s energy poverty and the last period’s energy poverty is assumed to affect this period’s health. Allowing for the dynamics of health and energy poverty within our joint modelling framework, we can explore persistence and inter-dependence. Thus, we will be able to provide important evidence on how health and energy poverty are related over time which can be used for policy making.

The rest of the paper is structured as follows. “Data and indicators” describes the data and variables used in the analysis. “Energy poverty and poor health in Australia: descriptive analysis” provides some descriptive statistics on the relationship between energy poverty and poor health in Australia. The empirical specification is detailed in “Econometric specification”. “Estimation results” presents the results; “Discussion and conclusion” concludes.

## Data and indicators

### Data and sample selection

To study the longitudinal relationship between energy poverty and poor health in Australia, we used a balanced panel over the period 2005–2018 from the Survey of Household, Income and Labour Dynamics of Australia (HILDA). The HILDA survey is a long household panel containing a wealth of information relevant for our aims, including data on health status, disposable income, energy expenditure and inability to meet some current financial obligations like paying energy bills. Interviews are conducted from mid-August (end of Australian winter) to early January (Australian summer) (Summerfield et al. 2019). Given its longitudinal nature, it allows us to effectively analyse the dynamics of energy poverty and poor health. In the analysis all variables are measured at the individual level.

### Health

We employ three measures of health in our analysis: general health and mental health as measured by the SF-36 and a measure of self-assessed health. The inter-related dynamics of health and energy poverty may be different for physical and mental health. We can also explore if our findings are robust across different aspects and ways of measuring health.

In all waves of HILDA Survey individuals’ general and mental well-being are measured using the SF-36 Health Survey (Ware et al. 2000), an internationally recognised multi-dimensional diagnostic tool for assessing functional health status and well-being. Multi-dimensional measures of health are recognized as being a more accurate representation of a person’s health compared to a single item measure of health (Horn et al. 2017). The scores for both measures range from 0 to 100. Where 0 is very poor health and 100 is the best health possible.

Our interest is in poor health. While there are no universally accepted threshold scores for defining poor general and mental health indicators, for the purposes of this paper, we will follow the approach used in the HILDA Statistical Reports (e.g., Wilkins 2017). Poor general health is defined as a score less than or equal to 37, on the basis that approximately 10% of the population are at or below this threshold. Similarly, poor mental health is defined as a score less than or equal to 52, on the basis that approximately 10% of the population are at or below this threshold. Note that, even if this may be seen as arbitrary, its use in previous publications using HILDA Survey data allows comparisons with those studies. We focus on dichotomous measures of health to ease interpretation as we can then clearly explore the differences between those with good vs poor health. Nonetheless, we tested the robustness of the thresholds chosen re-performing the analysis using alternative definitions of the cut-off points: less than 50 for general health (20% of the population). Our main results concerning energy poverty dynamics, and its relation to poor health, were essentially unaffected by the alternative cut-off points.

Self-assessed health is measured by a question asking respondents. ‘In general, how would you say your health is?’ The options are: (1) excellent; (2) very good; (3) good; (4) fair; and (5) poor. We generate a dummy variable which equals one if the individual reports poor health and is equal to zero otherwise.

### Energy poverty

Energy poor households are those that are unable to afford to adequately heat one’s home either because they cannot afford it or because of energy inefficiency issues associated with their home (Moore 2012). As such, energy poverty involves a “complex interaction of low income and energy efficiency” (Healy and Clinch 2002), with energy prices, individual energy needs and climatic conditions being important components (Boardman 2012; Bouzarovski et al. 2012; Liddell et al. 2012). Yet, while there is consensus around the concept, there is no single accepted definition of how to identify individuals in energy poverty (Moore 2012; Li et al. 2014).

In this paper, we adopt three measures of energy poverty commonly used in the literature with the hope of covering a wide range of people affected by this phenomenon. First, we rely on the objective energy poverty measure proposed by Hills (2012).^5^ Households are defined as energy poor when they meet the following two criteria: (1) their expenditures on fuel/energy exceed the median level of the energy expenditure of the reference population^6^; and (2) the household’s residual income (equivalized income after equivalized energy expenditure) is below the income poverty line of 60 per cent of the median income after housing costs. This means that after fuel expenditure the household’s disposable income is below the poverty threshold. We refer to this measure as [linchcost].

To equivalize income, we use the ‘modified OECD’ scale (Hagenaars et al. 1994), which divides household income by 1 for the first household member plus 0.5 for each other household member aged 15 or over, plus 0.3 for each child under 15. A family comprising two adults and two children under 15 years of age would, therefore, have an equivalence scale of 2.1 (1 + 0.5 + 0.3 + 0.3), meaning that the family would need to have an income 2.1 times that of a single-person household to achieve the same standard of living. This scale recognises that larger households require more income, but it also recognises that there are economies of scale in consumption (for example, the rent on a two-bedroom flat is typically less than twice the rent on an otherwise comparable one-bedroom flat) and that children require less resources than adults. The equivalised income calculated for a household is then assigned to each member of the household, the implicit assumption being that all household members experience the same standard of living (which will, of course, not always be the case—particularly in households containing unrelated people).

Further, following Thomson and Snell’s (2013) we consider two subjective indicators: inability to keep the home adequately warm [noheat], and inability to pay utility bills on time [nopaybills]. These are modelled as dummy variables that take the value 1 when the household reports that they cannot keep their home warm and cannot pay the bills on time, respectively. These measures control for a self-reported inadequacy to keep one’s home at a comfortable temperature or difficulty in paying one’s energy bills which can be perceived as being under financial stress (Breunig and Cobb-Clark 2005). Some households restrict their energy consumption to the detriment of their health or wellbeing but pay their energy bills and do not feel like they have difficulty in paying this bill because of lower costs from reduced consumption so may not self-report as being behind with their bills. This form of hardship is often hidden and there is relatively little empirical data on households such as these who are facing material deprivation, either directly as a result of reducing their energy consumption or indirectly through doing without other goods or services. For households that are in arrears with their energy bills, some of these may report no energy expenditure and not be recording in our first measure [linchcost]. Disconnection because of failure to pay for energy is becoming increasing in most Australian jurisdictions.

### Other controls

In all model specifications presented, we control for individual and household characteristics that are likely to influence both health and energy poverty. These factors are related to the social determinants of health which are likely to be associated with both energy poverty and health (Jessel et al. 2019). We also include household size and composition which will influence and be influenced by the social determinants of health. These include age, marital status, employment, educational attainment, equivalized household income, and number of dependent children. Descriptive statistics of these variables are presented in Table 1.

**Table 1:** Descriptive statistics (averaged over the sample period)

Variables	Mean (standard errors)
Age (mean)	45.8 (0.262)
Low education (%)	0.31 (0.006)
Medium education (%)	0.47 (0.006)
High education (%)	0.23 (0.007)
Household responsible employed (%)	0.71 (0.007)
Number of own dependent children (mean)	0.61 (0.013)
Married or de facto (%)	0.63 (0.006)
Household equivalised income (mean)	$47,948.88 (607.38)

^a^Balanced panel of 9672 individuals, for a total of 36,839 person-year observations

## Energy poverty and poor health in Australia: descriptive analysis

Table 2 presents descriptive statistics for the percentage of individuals reporting being in energy poverty for the three different indicators of energy poverty. Results show that 4.20% of the sample are classified as low income/high cost [linchcost], 10.89% report not being able to pay their bills on time [nopaybills], and 2.45% report not being able to adequately heat their homes [noheat]. The most common reported measure is being behind with bills. The small sample sizes for some of the indicators may impact on our ability to detect statistically significant results.

**Table 2:** Descriptive statistics: different definitions of energy poverty (sample size in brackets)

Energy poverty definitions	Population in energy poverty
	%
Linchcost	4.20 (125,736)
Nopaybills	10.89 (96,554)
Noheat	2.45 (96,343)

Table 3 shows the cross-tabulation of the different measures of energy poverty. 21.35% of the 4.20% of people who are classified as low income/high cost [linchost] also report being behind with bill [nopaybills]. 6.3% of the 4.20% who are [linchost] also report not adequately heating their home [noheat].

**Table 3:** Cross-tabulations between fuel poverty indicators

Indicator	Percentage of all households identified as energy poor by the two indicators	
	Nopaybills	Noheat
Linchcost	21.35	6.34
Nopaybills	–	13.78

Note: Estimates are equal to the average level of overlapping for the period 2005–2018

**Fig. 1:**
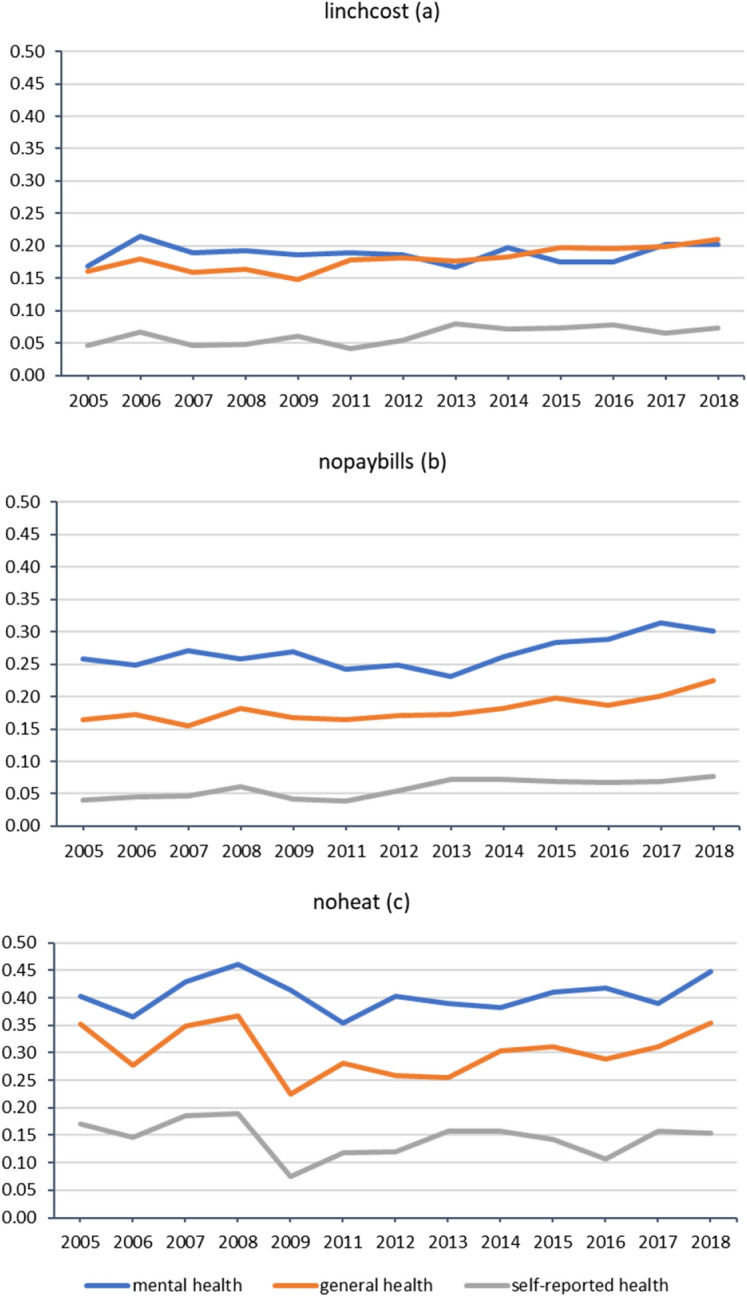
Percentage in poor general, mental and self-reported health by different definitions of energy poverty (*100): 2005–2018

Figure 1 graphically shows the dynamics of poor health across the 13 years of the survey among those who are classified as in energy poverty according to one of the three measures. The strongest relationship between poor health (all three types) and a measure of energy poverty is seen for those who report not to be able to adequately warm their home. For all measures except low income/high-cost individuals in energy poverty appear more likely to have poor mental health than general or physical health. We can also see a moderate upward trajectory over time.

Table 4 reports the proportion of the sample that experiences energy poverty, the length of the energy poverty spell, and how likely they are to be in poor health given the amount of time in energy poverty. The longer individuals have been energy poor, the more likely they are to report poor health. The increase is particularly significant for poor mental health.

**Table 4:** Percentage of individuals in poor health by number of years in energy poverty (*100): 2005–2018

Number of years in energy poverty	Linchcost			Nopaybills			Noheat^a^		
	M	G	S	M	G	S	M	G	S
1	0.18	0.16	0.06	0.25	0.16	0.06	0.39	0.27	0.12
2	0.20	0.18	0.06	0.28	0.19	0.07	0.42	0.33	0.13
3	0.21	0.21	0.08	0.28	0.18	0.07	0.37	0.32	0.20
4	0.25	0.17	0.10	0.36	0.23	0.10	0.41	0.29	0.24
5 or more	0.28	0.14	0.10	0.37	0.23	0.14			
Sample size	3130	3103	3104	8023	7952	7976	2108	2081	2097

*M* poor mental health, *G* poor general health, *S* poor self-reported health

^a^Few cases were found of individuals energy poor [noheat] for 5 years or more, so we recoded this group together with 4 years

In the next section of the paper, we further explore the relationship we observed in the descriptive statistics showing some evidence of state dependence and a bi-directional relationship between energy poverty and health. Disentangling this relationship will be crucial for understanding the dynamics of the two outcomes and for the design of policy. If there are spillover effects between health and energy poverty, policies aimed at pulling individuals out of energy poverty may contribute to improve individuals’ health status breaking the cycle and ultimately reducing costs for governments. This also has important implications for other health economics research trying to estimate causal relationships between health and various dimensions of poverty.

## Econometric specification

To understand the dynamics of energy poverty and health, we start with simple dynamic univariate probit models (i.e., separate models for energy poverty and health). Models are estimated separately for each of the health outcomes for both the energy poverty and health equations. This allows us to test the restrictions imposed by the univariate models on the relationship between energy poverty and health:

1 $${H}_{it}={x}_{it}^{{\prime}}{\beta }_{1}+ {H}_{it-1}{{\beta }_{12}+{EP}_{it-1}{\beta }_{14}+ u}_{1it},$$

2 $${EP}_{it}={x}_{it}^{{\prime}}{\beta }_{2}+{EP}_{it-1}{\beta }_{21}+{H}_{it-1}{\beta }_{24}+{u}_{2it}.$$

The dependent variables are the dummy indicators ${H}_{1it}$ (equal to one if the individual is classified as being in poor health as measured by one of our three health outcome measures in *t*, and zero otherwise) and ${EP}_{2it}$ (equal to one if *i* is in energy poverty as reported by one of the three measures of energy poverty in *t*, and zero otherwise). ${x}_{it}$ is a vector of independent variables such as age, dummies for basic and some higher education (degree qualifications or higher is the reference category), marital status (= 1 if married or cohabiting). Household-level characteristics are also included: equivalized household income, a dummy for employment status, and the number of household-dependent children (< 15 year-old). These variables can be time-varying. Year dummies are included in all specifications to control for the wider macroeconomic environment. Structural differences in local conditions (e.g., differences in regional prices and regional energy policies) are controlled for by including regional dummies in all models. ${H}_{1it-1}$ and ${EP}_{2it-1}$ are lagged health and energy poverty, respectively. In both equations (poor health and energy poverty), the same explanatory variables are used. The error terms ${u}_{1it}$ and ${u}_{2it}$ are assumed to be independent over time and to follow a bivariate normal distribution, with zero means, unit variances. The error term consists of a random component ${e}_{it}$ which can vary over time and an individual specific time-constant component, ${\delta }_{i}$.

To account for initial conditions—the fact that we are only observing health and energy poverty for a short period of time for individuals in the sample—, we follow Devicienti and Poggi (2011) who extend to the univariate case the simple approach proposed by Wooldridge (2005) to account for the fact that we do not observe health and energy poverty across an individual’s entire life. This method is operationalized through a Conditional Maximum Likelihood (CML) estimator that allows the mean of the random effects appearing in the poor health and energy poor equations to depend on the initial observations in the data of both the individual’s energy poor and poor health statuses and the observed history of strictly explanatory variables.^7^

We specify the individual specific effects ${c}_{i1}$ and ${c}_{i2}$ given the initial energy poverty observation,${y}_{1i1}$, and the initial poor health observation, ${y}_{2i1}$ and take the mean of the time-constant explanatory variables ${\overline{x} }_{i}$ as follows:

3 $${c}_{1i}={a}_{10}+{a}_{11}{H}_{i1}+{a}_{12}{EP}_{i1}+{\overline{x} }_{i}^{{\prime}}{a}_{13}+{\alpha }_{1i},$$

4 $${c}_{2i}={a}_{20}+{a}_{21}{H}_{i1}+{a}_{22}{EP}_{i1}+{\overline{x} }_{i}^{{\prime}}{a}_{23}+{\alpha }_{2i},$$

where ${a}_{j0}$, ${a}_{j1}$, ${a}_{j2,}$ and ${a}_{j3}$ (*j* = 1, 2) are parameters to be estimated, (${\alpha }_{1i},$ ${\alpha }_{2i}$) which are normally distributed with covariance matrix ∑_α_

5 $$\sum_{{\upalpha }} = \left( {\begin{array}{*{20}c} {\sigma_{\alpha 1}^{2} } & {\sigma_{\alpha 1} \sigma_{\alpha 2} \rho_{c} } \\ . & {\sigma_{\alpha 2}^{2} } \\ \end{array} } \right).$$

Then after inserting in Eqs. (1) and (2) we end up with:

6 $${H}_{it}^{*}={x}_{it}^{{\prime}}{\beta }_{1}+{H}_{i,t-1}{\gamma }_{11}+{EP}_{i,t-1}{\gamma }_{12}+{ a}_{10}+{\overline{x} }_{i}^{{\prime}}{a}_{13}+{\alpha }_{1i}+{u}_{1it},$$

7 $${EP}_{2it}^{*}={x}_{it}^{{\prime}}{\beta }_{2}+{H}_{i,t-1}{\gamma }_{21}+{EP}_{i,t-1}{\gamma }_{22}+ {a}_{20}+{\overline{x} }_{i}^{{\prime}}{a}_{23}+{\alpha }_{2i}+{u}_{2it}.$$

This approach requires a balanced panel of respondents who are present in all waves included in the analysis using a balanced panel, we could potentially exacerbate attrition and sample selection present in the data. In fact, this is not the case, since Wooldridge’s method has some advantages in facing selection and attrition problems. As explained in Wooldridge (2005, p. 44), it allows selection and attrition to depend on the initial conditions and, therefore, it allows attrition to differ across initial levels of the outcome variables. Individuals with different initial statuses can have different missing data probabilities. Thus, we consider selection and attrition without explicitly modelling them as a function of the initial conditions. As a result, the analysis is less complicated, and it compensates for the potential loss of information from using a balanced panel. Moreover, in the conditional MLE we can ignore any stratification that is a function of the initial level of deprivation and of the time-constant explanatory variables: thus, using sampling weights would lead to an efficiency loss.

Finally, to disentangle the relationship energy poverty and health, we estimate a dynamic random-effect bivariate probit model. This model builds upon the univariate probit by allowing for correlated unobserved heterogeneity between health and energy poverty. Similar to the univariate framework we also control for initial conditions, state and cross-dependence for health and energy poverty.

This can be expressed by the following formulae:

8 $${H}_{it}^{*}={x}_{it}^{{\prime}}{\beta }_{1}+{H}_{i,t-1}{\gamma }_{11}+{EP}_{i,t-1}{\gamma }_{12}+{c}_{1i}+{u}_{1it},$$

9 $${EP}_{it}^{*}={x}_{it}^{{\prime}}{\beta }_{2}+{H}_{i,t-1}{\gamma }_{21}+{EP}_{i,t-1}{\gamma }_{22}+{c}_{2i}+{u}_{2it},$$

10 $${{H}_{it}^{*}, EP}_{it}^{*}=1 \left[{{H}_{it}^{*}, EP}_{it}^{*}>0\right]\quad t=2,\dots ,T.$$

Unlike in the univariate specification in Eqs. (1) and (2), in Eqs. (8) to (10), the error terms ${u}_{1it}$ and ${u}_{2it}$ are assumed to have cross-equation covariance$\rho$. The model also includes individual random effects, ${c}_{1i}$ and ${c}_{2i}$ assumed to be bivariate normal with variances ${\sigma }_{c1}^{2}$ and ${\sigma }_{c2}^{2}$ and covariances${\sigma }_{c1}{\sigma }_{c2}{\rho }_{c}$. The model assumes that (${c}_{1i}$, ${c}_{2i}$), (${u}_{1it}$, ${ u}_{2it}$; *t* = 1,..., *T*) and (${x}_{it}$; *t* = 1,..., *T*) are independent. The dynamics of the model is assumed to be of first order for simplicity. However, as a robustness check we test this assumption by exploring second order dynamics.

## Estimation results

### Univariate random-effects dynamics models for energy poverty and poor health

We start with probit models estimated separately for the probability of being in energy poverty for the three different ways of measuring health (Tables 5, 6, and 7) and the probability of being in poor health for the three health outcomes in Tables 8, 9 and 10. In the basic pooled probit framework we start by assuming initial conditions do not impact on the findings (columns 1, 3 and 5 in each Table). Next, we add initial conditions to understand how endogeneity in the lagged health status and fuel poverty are likely to impact on our results (columns 2, 4 and 6 in each Table).

**Table 5:** Estimates of the probit model for the probability of being in energy poverty with health measured by SF-36: General health

Def. of fuel poverty	No pay bills		No heat		Low income/high cost	
	Pooled probit model	Initial conditions: Wooldridge	Pooled probit model	Initial conditions: Wooldridge	Pooled probit model	Initial conditions: Wooldridge
Poor health (*t* − 1)	0.30***	0.15^***^	0.39^***^	0.32^***^	0.13^***^	0.064
General health	(0.033)	(0.053)	(0.050)	(0.085)	(0.038)	(0.055)
Fuel poor (*t* − 1)	1.76***	0.82^***^	1.96^***^	0.88^***^	1.03^***^	0.62^***^
	(0.026)	(0.037)	(0.055)	(0.079)	(0.037)	(0.047)
Poor health (1)		0.29^***^		0.22^*^		0.12^*^
General health		(0.079)		(0.125)		(0.066)
Fuel poor (1)		1.36^***^		1.72^***^		0.54^***^
		(0.064)		(0.196)		(0.068)
Age	− 0.011^***^	− 0.018^***^	− 0.0071^***^	− 0.012^***^	0.00023	− 0.0011
	(0.001)	(0.002)	(0.001)	(0.003)	(0.001)	(0.002)
Medium education	0.036	0.068	0.075^*^	0.12	− 0.037	− 0.032
	(0.026)	(0.051)	(0.043)	(0.086)	(0.031)	(0.043)
High education	− 0.17^***^	− 0.10	− 0.15^**^	− 0.039	− 0.16^***^	− 0.097
	(0.033)	(0.068)	(0.063)	(0.123)	(0.043)	(0.061)
Married/cohabiting	− 0.11^***^	− 0.079	− 0.37^***^	− 0.45^***^	0.29^***^	0.10
	(0.027)	(0.056)	(0.043)	(0.106)	(0.036)	(0.077)
Head of household employed	0.053^*^	0.022	− 0.18^***^	− 0.23^***^	− 0.27^***^	− 0.24^***^
	(0.032)	(0.052)	(0.051)	(0.088)	(0.040)	(0.056)
Number of own dependent children	0.057^***^	0.047^**^	0.046^**^	− 0.035	0.14^***^	0.17^***^
	(0.011)	(0.024)	(0.020)	(0.047)	(0.013)	(0.030)
Household income (log)	− 0.37^***^	− 0.25^***^	− 0.32^***^	− 0.15^***^	− 1.02^***^	− 1.08^***^
	(0.020)	(0.035)	(0.024)	(0.059)	(0.044)	(0.031)
Year dummies	Yes	Yes	Yes	Yes	Yes	Yes
Regional dummies	Yes	Yes	Yes	Yes	Yes	Yes
Longitudinal average variables	No	Yes	No	Yes	No	Yes
Constant	2.59^***^	7.46^***^	1.64^***^	9.90^***^	8.59^***^	12.4^***^
	(0.220)	(0.745)	(0.266)	(1.376)	(0.456)	(0.695)
${\sigma }_{\alpha }$		− 0.45^***^		− 0.10		− 1.33^***^
		(0.075)		(0.138)		(0.129)
Observations	38,610	38,610	37,972	37,972	49,093	49,093
Log-likelihood	− 7565.4	− 6784.1	− 2303.9	− 2061.9	− 4863.6	− 4718.1

Notes: Robust SEs are displayed, to account for individual repeated observations in the panel

^*^p < 0.10, ^**^p < 0.05, ^***^p < 0.01

**Table 6:** Estimates of the probit model for the probability of being in energy poverty with health measured by self-assessed health

Def. of fuel poverty	No pay bills		No heat		Low income/high cost	
	Pooled probit model	Initial conditions: Wooldridge	Pooled probit model	Initial conditions: Wooldridge	Pooled probit model	Initial conditions: Wooldridge
Poor health (*t* − 1)	0.27^***^	0.11	0.42^***^	0.26^**^	0.24^***^	0.18^**^
Self-reported health	(0.061)^***^	(0.085)	(0.077)	(0.121)	(0.058)	(0.078)
Fuel poor (*t* − 1)	1.78^***^	0.78^***^	2.02^***^	0.87^***^	1.04^***^	0.61^***^
	(0.026)	(0.037)	(0.054)	(0.079)	(0.037)	(0.047)
Poor health (1)		0.27^*^		0.052	0.00063	0.20^*^
General health		(0.160)		(0.227)	(0.001)	(0.109)
Fuel poor (1)		1.47^***^		1.91^***^	− 0.049	0.58^***^
		(0.067)		(0.194)	(0.030)	(0.067)
Age	− 0.011^***^	− 0.018^***^	− 0.0061^***^	− 0.011^***^	− 0.15^***^	0.000091
	(0.001)	(0.002)	(0.001)	(0.003)	(0.043)	(0.002)
Medium education	0.029	0.064	0.080^*^	0.13	0.30^***^	− 0.040
	(0.026)	(0.053)	(0.042)	(0.085)	(0.036)	(0.042)
High education	− 0.18^***^	− 0.17^**^	− 0.11^*^	− 0.00017	− 0.27^***^	− 0.079
	(0.034)	(0.072)	(0.061)	(0.123)	(0.040)	(0.062)
Married/cohabiting	− 0.14^***^	− 0.13^**^	− 0.41^***^	− 0.42^***^	0.13^***^	0.10
	(0.027)	(0.057)	(0.042)	(0.104)	(0.014)	(0.078)
Head of household employed	0.023	0.0046	− 0.14^***^	− 0.14	− 1.01^***^	− 0.24^***^
	(0.032)	(0.052)	(0.051)	(0.087)	(0.043)	(0.055)
Number of own dependent children	0.064^***^	0.063^***^	0.032	− 0.059	0.00063	0.15^***^
	(0.011)	(0.024)	(0.020)	(0.048)	(0.001)	(0.031)
Household income (log)	− 0.37^***^	− 0.27^***^	− 0.32^***^	− 0.17^***^	− 0.049	− 1.07^***^
	(0.021)	(0.036)	(0.025)	(0.060)	(0.030)	(0.031)
Year dummies	Yes	Yes	Yes	Yes	Yes	Yes
Regional dummies	Yes	Yes	Yes	Yes	Yes	Yes
Longitudinal average variables	No	Yes	No	Yes	No	Yes
Constant	2.68^***^	6.74^***^	1.77^***^	9.36^***^	8.40^***^	12.1^***^
	(0.233)	(0.752)	(0.274)	(1.359)	(0.448)	(0.696)
${\sigma }_{\alpha }$		− 0.37^***^		− 0.093		− 1.28^***^
		(0.074)		(0.136)		(0.126)
Observations	38,038	38,038	37,477	37,477	48,510	48,510
Log-likelihood	− 7537.6	− 6687.2	− 2383.4	− 2118.5	− 4967.4	− 4807.0

Notes: Robust SEs are displayed, to account for individual repeated observations in the panel

^*^p < 0.10, ^**^p < 0.05, ^***^p < 0.01

**Table 7:** Estimates of the probit model for the probability of being in energy poverty with health measured by SF-36: mental health

Def. of fuel poverty	No pay bills		No heat		Low income/high cost	
	Pooled probit model	Initial conditions: Wooldridge	Pooled probit model	Initial conditions: Wooldridge	Pooled probit model	Initial conditions: Wooldridge
Poor health (*t* − 1)	0.29^***^	0.18^***^	0.35^***^	0.20^***^	0.045	0.017
Mental health	(0.031)	(0.042)	(0.045)	(0.068)	(0.037)	(0.049)
Fuel poor (*t* − 1)	1.74^***^	0.79^***^	1.95^***^	0.87^***^	1.03^***^	0.61^***^
	(0.025	(0.036)	(0.053)	(0.075)	(0.035)	(0.045)
Poor health (1)		0.16^**^		0.40^***^		− 0.0043
General health		(0.067)		(0.101)		(0.059)
Fuel poor (1)		1.38^***^		1.78^***^		0.53^***^
		(0.063)		(0.180)		(0.064)
Age	− 0.010^***^	− 0.017^***^	− 0.0057^***^	− 0.0099^***^	− 0.000032	− 0.0017
	(0.001)	(0.002)	(0.001)	(0.003)	(0.001)	(0.002)
Medium education	0.035	0.076	0.060	0.14^*^	− 0.042	− 0.034
	(0.025)	(0.050)	(0.041)	(0.081)	(0.029)	(0.040)
High education	− 0.17^***^	− 0.12^*^	− 0.16^***^	− 0.020	− 0.15^***^	− 0.087
	(0.032)	(0.067)	(0.060)	(0.116)	(0.041)	(0.058)
Married/cohabiting	− 0.11^***^	− 0.083	− 0.36^***^	− 0.40^***^	0.29^***^	0.14^*^
	(0.026)	(0.055)	(0.041)	(0.102)	(0.034)	(0.073)
Head of household employed	0.055^*^	0.029	− 0.17^***^	− 0.21^**^	− 0.29^***^	− 0.26^***^
	(0.031)	(0.050)	(0.048)	(0.084)	(0.038)	(0.053)
Number of own dependent children	0.061^***^	0.062^***^	0.032^*^	− 0.044	0.13^***^	0.16^***^
	(0.011)	(0.023)	(0.019)	(0.046)	(0.013)	(0.029)
Household income (log)	− 0.36^***^	− 0.25^***^	− 0.32^***^	− 0.17^***^	− 1.01^***^	− 1.06^***^
	(0.020)	(0.034)	(0.023)	(0.055)	(0.041)	(0.029)
Year dummies	Yes	Yes	Yes	Yes	Yes	Yes
Regional dummies	Yes	Yes	Yes	Yes	Yes	Yes
Longitudinal average variables	No	Yes	No	Yes	No	Yes
Constant	2.47^***^	7.14^***^	1.60^***^	9.16^***^	8.49^***^	12.5^***^
	(0.220)	(0.715)	(0.259)	(1.293)	(0.426)	(0.665)
${\sigma }_{\alpha }$		− 0.43^***^		− 0.15		− 1.30^***^
		(0.072)		(0.133)		(0.120)
Observations	41,773	41,773	40,524	40,524	52,866	
Log-likelihood	− 8083.6	− 7238.6	− 2522.9	− 2250.0	− 5444.0	− 5282.8

Notes: Robust SEs are displayed, to account for individual repeated observations in the panel

^*^p < 0.10, ^**^p < 0.05, ^***^p < 0.01

**Table 8:** Estimates of the probit model for the probability of being in poor general health as measured by the SF-36 using different definitions of fuel poverty

	No pay bills		No heat		Low income/high cost	
	Pooled probit model	Initial conditions: Wooldridge	Pooled probit model	Initial conditions: Wooldridge	Pooled probit model	Initial conditions: Wooldridge
Poor health (*t* − 1)	2.13^***^	0.81^***^	2.15^***^	0.81^***^	2.14^***^	0.77^***^
General health	(0.025)	(0.039)	(0.026)	(0.040)	(0.022)	(0.035)
Fuel poor (*t* − 1)	0.30^***^	0.17^***^	0.34^***^	0.040	0.043	− 0.0013
	(0.037)	(0.054)	(0.066)	(0.097)	(0.046)	(0.064)
Poor health (1)		2.22^***^		2.27^***^		2.32^***^
General health		(0.096)		(0.098)		(0.085)
Fuel poor (1)		0.30^***^		0.62^***^		− 0.0015
		(0.091)		(0.226)		(0.124)
Age	0.0049^***^	0.0048^*^	0.0040^***^	0.0045^*^	0.0036^***^	0.0038^*^
	(0.001)	(0.003)	(0.001)	(0.003)	(0.001)	(0.002)
Medium education	− 0.063^**^	− 0.083	− 0.074^***^	− 0.11	− 0.040^*^	− 0.039
	(0.026)	(0.067)	(0.026)	(0.068)	(0.022)	(0.059)
High education	− 0.035	− 0.010	− 0.059^*^	− 0.062	− 0.051^*^	− 0.012
	(0.031)	(0.082)	(0.031)	(0.083)	(0.027)	(0.074)
Married/cohabiting	− 0.049^*^	− 0.077	− 0.030	− 0.056	− 0.064^***^	− 0.081
	(0.026)	(0.068)	(0.027)	(0.069)	(0.022)	(0.060)
Head of household employed	− 0.16^***^	− 0.075	− 0.16^***^	− 0.088^*^	− 0.20^***^	− 0.13^***^
	(0.029)	(0.052)	(0.030)	(0.052)	(0.025)	(0.045)
Number of own dependent children	− 0.039^***^	0.047	− 0.034^***^	0.059^*^	− 0.039^***^	0.031
	(0.013)	(0.030)	(0.013)	(0.030)	(0.012)	(0.027)
Household income (log)	− 0.088^***^	− 0.019	− 0.098^***^	− 0.0088	− 0.10^***^	− 0.0024
	(0.020)	(0.039)	(0.020)	(0.039)	(0.017)	(0.033)
Year dummies	Yes	Yes	Yes	Yes	Yes	Yes
Regional dummies	Yes	Yes	Yes	Yes	Yes	Yes
Longitudinal average variables	No	Yes	No	Yes	No	Yes
Constant	− 0.78^***^	0.33	− 0.63^***^	0.92	− 0.51^***^	1.44^*^
	(0.221)	(0.827)	(0.223)	(0.824)	(0.192)	(0.738)
${\sigma }_{\alpha }$		0.21^***^		0.23^***^		0.27^***^
		(0.072)		(0.072)		(0.062)
Observations	38,610	38,610	37,972	37,972	49,093	49,093
*Log-likelihood*	− 7652.2	− 6585.1	− 7506.5	− 6435.9	− 10,184.8	− 8701.05

Notes: Robust SEs are displayed, to account for individual repeated observations in the panel

^*^p < 0.10, ^**^p < 0.05, ^***^p < 0.01

**Table 9:** Estimates of the probit model for the probability of being in poor self-assessed health using different definitions of fuel poverty

	No pay bills		No heat		Low income/high cost	
	Pooled probit model	Initial conditions: Wooldridge	Pooled probit model	Initial conditions: Wooldridge	Pooled probit model	Initial conditions: Wooldridge
Poor health (*t* − 1)	2.21^***^	0.93^***^	2.23^***^	0.94^***^	2.23^***^	0.91^***^
Self-assessed health	(0.047)	(0.071)	(0.047)	(0.072)	(0.039)	(0.059)
Fuel poor (*t* − 1)	0.31^***^	0.13	0.35^***^	− 0.070	0.099	0.025
	(0.052)	(0.079)	(0.084)	(0.127)	(0.062)	(0.085)
Poor health (1)		2.16^***^		2.17^***^		2.28^***^
Self− assessed Health		(0.186)		(0.186)		(0.158)
Fuel poor (1)		0.53^***^		0.69^***^		0.068
		(0.113)		(0.240)		(0.145)
Age	0.0037^***^	0.00082	0.0026^*^	− 0.00075	0.0026^**^	0.0012
	(0.001)	(0.003)	(0.001)	(0.003)	(0.001)	(0.003)
Medium education	− 0.061	− 0.049	− 0.073^*^	− 0.065	− 0.052	− 0.014
	(0.038)	(0.086)	(0.038)	(0.086)	(0.032)	(0.074)
High education	− 0.14^***^	− 0.14	− 0.18^***^	− 0.20^*^	− 0.18^***^	− 0.16
	(0.050)	(0.117)	(0.051)	(0.118)	(0.045)	(0.105)
Married/cohabiting	− 0.078^**^	− 0.0044	− 0.042	− 0.034	− 0.088^***^	0.016
	(0.038)	(0.107)	(0.040)	(0.109)	(0.033)	(0.092)
Head of household employed	− 0.29^***^	− 0.18^**^	− 0.29^***^	− 0.19^**^	− 0.28^***^	− 0.16^**^
	(0.045)	(0.081)	(0.046)	(0.082)	(0.039)	(0.069)
Number of own dependent children	− 0.047^**^	0.021	− 0.050^**^	0.023	− 0.045^**^	− 0.018
	(0.022)	(0.053)	(0.022)	(0.054)	(0.019)	(0.046)
Household income (log)	− 0.12^***^	0.00092	− 0.14^***^	0.00075	− 0.14^***^	− 0.0064
	(0.026)	(0.059)	(0.026)	(0.059)	(0.021)	(0.049)
Year dummies	Yes	Yes	Yes	Yes	Yes	Yes
Regional dummies	Yes	Yes	Yes	Yes	Yes	Yes
Longitudinal average variables	No	Yes	No	Yes	No	Yes
Constant	− 0.84^***^	1.77	− 0.55^*^	2.95^***^	− 0.53^**^	3.12^***^
	(0.293)	(1.150)	(0.291)	(1.146)	(0.238)	(1.020)
${\sigma }_{\alpha }$		0.13		0.13		0.17
		(0.122)		(0.125)		(0.104)
Observations	38,038	38,038	37,477	37,477	48,510	48,510
*Log-likelihood*	− 2956.3	− 2605.4	− 2889.3	− 2546.4	− 4115.5	− 3602.3

Notes: Robust SEs are displayed, to account for individual repeated observations in the panel

^*^p < 0.10, ^**^p < 0.05, ^***^p < 0.01

**Table 10:** Estimates of the probit model for the probability of being in poor mental health as measured by the SF-36 using different definitions of fuel poverty

	No pay bills		No heat		Low income/high cost	
	Pooled probit model	Initial conditions: Wooldridge	Pooled probit model	Initial conditions: Wooldridge	Pooled probit model	Initial conditions: Wooldridge
Poor health (*t* − 1)	1.56^***^	0.62^***^	1.58^***^	0.62^***^	1.60^***^	0.60^***^
Mental health	(0.023)	(0.032)	(0.023)	(0.032)	(0.019)	(0.028)
Fuel poor (*t* − 1)	0.29^***^	0.14^***^	0.37^***^	0.12	0.11^***^	0.085
	(0.029)	(0.042)	(0.058)	(0.075)	(0.041)	(0.052)
Poor health (1)		1.28^***^		1.31^***^		1.37^***^
General Health		(0.062)		(0.063)		(0.055)
Fuel poor (1)		0.21^***^		0.60^***^		0.018
		(0.066)		(0.155)		(0.092)
Age	− 0.0066^***^	− 0.011^***^	− 0.0070^***^	− 0.011^***^	− 0.0079^***^	− 0.013^***^
	(0.001)	(0.002)	(0.001)	(0.002)	(0.001)	(0.002)
Medium education	− 0.087^***^	− 0.065	− 0.094^***^	− 0.083^*^	− 0.067^***^	− 0.048
	(0.022)	(0.049)	(0.022)	(0.049)	(0.019)	(0.043)
High education	− 0.032	0.061	− 0.050^*^	0.030	− 0.072^***^	0.014
	(0.027)	(0.060)	(0.027)	(0.060)	(0.024)	(0.055)
Married/cohabiting	− 0.13^***^	− 0.20^***^	− 0.12^***^	− 0.20^***^	− 0.14^***^	− 0.18^***^
	(0.022)	(0.049)	(0.023)	(0.049)	(0.019)	(0.044)
Head of household employed	− 0.17^***^	− 0.10^**^	− 0.16^***^	− 0.12^***^	− 0.19^***^	− 0.12^***^
	(0.026)	(0.043)	(0.026)	(0.043)	(0.022)	(0.037)
Number of own dependent children	0.00052	0.060^***^	0.0092	0.070^***^	0.0016	0.054^***^
	(0.010)	(0.022)	(0.010)	(0.022)	(0.009)	(0.019)
Household income (log)	− 0.11^***^	0.022	− 0.12^***^	0.031	− 0.12^***^	0.0054
	(0.018)	(0.032)	(0.017)	(0.032)	(0.015)	(0.027)
Year dummies	Yes	Yes	Yes	Yes	Yes	Yes
Regional dummies	Yes	Yes	Yes	Yes	Yes	Yes
Longitudinal average variables	No	Yes	No	Yes	No	Yes
Constant	0.17	2.64^***^	0.28	2.90^***^	0.31^*^	2.87^***^
	(0.192)	(0.622)	(0.191)	(0.623)	(0.164)	(0.559)
${\sigma }_{\alpha }$		− 0.27^***^		− 0.25^***^		− 0.19^***^
		(0.061)		(0.061)		(0.052)
Observations	41,173	41,773	40,524	40,524	52,866	52,866
*Log-likelihood*	− 10,621.5	− 9565.2	− 10,469.1	− 9392.3	− 14,035.7	− 12,523.2

Notes: Robust SEs are displayed, to account for individual repeated observations in the panel

^*^p < 0.10, ^**^p < 0.05, ^***^p < 0.01

In columns (1, 3, and 5) in Table 5 we can see, across the three different measures of fuel poverty, that poor health in the previous period is significantly associated with the likelihood of being in fuel poverty. Similarly, being in fuel poverty in the previous period is significantly associated with the likelihood of being in fuel poverty in the current period for all three measures of fuel poverty. Once, we control for initial conditions (columns 2, 4 and 6 in Table 5), past poor general health is only statistically significant for the subjective measures of fuel poverty (no pay bills and no heat) but not for low income/high cost definition. Whereas the coefficient on past fuel poverty is reduced for all three fuel poverty measure suggesting that endogeneity bias may be impacting on the lagged coefficient in the naïve specification.

In Table 6, where health is defined as self-assessed health, the lagged health status variable is statistically significant in the naïve specification in columns 1, 3, and 5. The coefficients on lagged fuel poverty are similar to those found in Table 5 and are statistically significant in both the naïve specification in columns 1, 3, and 5 and in the second columns 2, 4, and 6 where we control for initial conditions albeit with coefficients of a smaller magnitude in the models controlling for initial conditions for no heat and low income/high cost only.

Finally, in Table 7 where we measure poor health using mental health from the SF-36, the lagged coefficients on fuel poverty across the naïve and models controlling for initial conditions are similar to those in Tables 5 and 6. However, when poor health is measured by mental health, lagged health status is no longer significant for energy poverty measured by low income/high cost in the naïve specification. Lagged poor mental health is statistically significant for the subjective measures of energy poverty for the naïve specification and when controlling for initial conditions.

Across Tables 5, 6, and 7, looking at the other coefficients in the model, a higher household income is significantly associated with a lower likelihood of being in all three measures of fuel poverty. Having more children significantly increases the likelihood of reporting all three measures. Being married or cohabiting compared to being single decreases the likelihood of reporting the two subjective measures of energy poverty but increases the likelihood of reporting the objective measure (low income/ high cost).

Next, we report on probability of being in poor health for the three health outcomes (Tables 8, 9, 10). Regarding the likelihood of reporting poor general health (Table 8), in our naïve specification where we do not control for initial conditions, fuel poverty in the previous period is associated with the likelihood of poor general health only for the subjective measures (nopaybills and noheat). When we control for initial conditions in columns 2, 4, and 6 of Table 8, the coefficient on lagged health is smaller but still significant suggesting the coefficients in the naïve estimated are likely to be biased. However, lagged fuel poverty is only significant for being behind with bill payments.

In Table 9, where we measure health using self-assessed health, the lagged health coefficient in both the naïve and Wooldridge specification with initial conditions are similar to those found in Table 8. Lagged fuel poverty in the naïve specification is statistically significant for the two subjective health measures. Once, we control for initial conditions lagged fuel poverty no longer statistically significant for any measure.

Last, in Table 10, where we measure poor health using the SF-36 mental health measure, the lagged health coefficients in both the naïve and Wooldridge specification are similar to those in Tables 8 and 9. Lagged energy poverty is statistically significant for all three energy poverty measures in the naïve specification. Once we control for initial conditions, the only energy poverty measure that is still significant is being behind with bill payments.

These basic probit models in Tables 5, 6, 7, 8, 9, and 10 suggest that there is some evidence of state dependency between energy poverty and poor health which is dependent upon how both energy poverty and health are measured. Across all measures of energy poverty and health there is stronger evidence for a cross-dependent relationship for subjective energy poverty measures than the objective measure. This will be explored further using a bivariate model allowing for correlated errors between health and energy poverty.

### Dynamic bivariate random-effects models for health and energy poverty

Tables 11, 12, and 13 report the estimates of the dynamic bivariate probit models for energy poverty and poor health, which relax the assumption of independence in the errors and the random effects of the two equations. Specification I report the estimated coefficients of a pooled dynamic bivariate probit model. Specification II report instead the estimated coefficients and SEs of the dynamic bivariate probit model with random effects and initial conditions employing the Wooldridge method.

**Table 11:** Dynamic bivariate probit: health measured as general health using the SF-36 for all three measures of energy poverty

	Nopaybills		Noheat		Linchcost	
	I	II	I	II	I	II
*Poor health*						
Poor health (*t* − 1)	2.13^***^	0.83^***^	2.15^***^	0.83^***^	2.14^***^	0.78^***^
Fuel poor (*t* − 1)	0.30^***^	0.13^**^	0.34^***^	0.014	0.091^**^	0.069
Poor health (1)		2.13^***^		2.24^***^		2.31^***^
Fuel poor (1)		0.32^***^		0.70^***^		− 0.026
Age	0.0049^***^	0.0022	0.0040^***^	0.0046^*^	0.0036^***^	0.0041^*^
Medium education	− 0.063^**^	− 0.069	− 0.074^***^	− 0.074	− 0.039^*^	− 0.073
High education	− 0.035	0.015	− 0.059^*^	− 0.018	− 0.049^*^	− 0.014
Married/cohabiting	− 0.049^*^	− 0.079	− 0.030	− 0.059	− 0.066^***^	− 0.082
Household resp. employed	− 0.16^***^	− 0.078	− 0.16^***^	− 0.085	− 0.20^***^	− 0.12^***^
# dependent children	− 0.039^***^	0.046	− 0.034^***^	0.059^**^	− 0.041^***^	0.030
Hhold income (log)	− 0.088^***^	− 0.020	− 0.098^***^	− 0.0070	− 0.096^***^	− 0.0025
Constant	− 0.78^***^	0.43	− 0.63^***^	0.56	− 0.57^***^	1.55^**^
*Energy poverty*						
Poor health (*t* − 1)	0.30^***^	0.12^**^	0.39^***^	0.29^***^	0.13^***^	0.084
Fuel poor (*t* − 1)	1.76^***^	0.84^***^	1.96^***^	0.98^***^	1.05^***^	0.62^***^
Poor health (1)		0.27^***^		0.23^**^		0.100^*^
Fuel poor (1)		1.32^***^		1.60^***^		0.49^***^
Age	− 0.011^***^	− 0.018^***^	− 0.007^***^	− 0.012^***^	− 0.0010	− 0.0021
Medium education	0.036	0.060	0.075^*^	0.18^**^	− 0.029	− 0.033
High education	− 0.17^***^	− 0.090	− 0.15^**^	− 0.0044	− 0.15^***^	− 0.11^**^
Married/cohabiting	− 0.11^***^	− 0.080	− 0.37^***^	− 0.44^***^	0.30^***^	0.079
Household resp. employed	0.053^*^	0.023	− 0.18^***^	− 0.21^**^	− 0.16^***^	− 0.17^***^
# dependent children	0.057^***^	0.044^*^	0.046^**^	− 0.037	0.15^***^	0.20^***^
Hhold income (log)	− 0.37^***^	− 0.25^***^	− 0.32^***^	− 0.15^**^	− 1.04^***^	− 1.11^***^
Constant	2.59^***^	7.25^***^	1.64^***^	9.19^***^	9.04^***^	13.2^***^
Year dummies	Yes	Yes	Yes	Yes	Yes	Yes
Regional dummies	Yes	Yes	Yes	Yes	Yes	Yes
Longitudinal average vars	No	Yes	No	Yes	No	Yes
Ρ		0.059^*^		0.067		0.055^*^
${\sigma }_{\alpha 1}$		0.80^***^		0.88^***^		0.56^***^
${\sigma }_{\alpha 2}$		1.12^***^		1.14^***^		1.17^***^
${\rho }_{\alpha }$		0.15^***^		0.11^*^		0.065
*Log-likelihood*	− 15,204.3	− 13,386	− 9805.4	− 9292.0	− 15,044.2	− 15,668
Observations	38,610	38,610	37,972	37,972	49,093	49,093

**Table 12:** Dynamic bivariate pooled probit model: health measured using self-assessed health and all three measures of energy poverty

	Nopaybills		Noheat		Linchcost	
	I	II	I	II	I	II
*Poor health*						
Poor health (*t* − 1)	1.86^***^	0.70^***^	1.87^***^	0.69^***^	1.87^***^	0.68^***^
Fuel poor (*t* − 1)	0.28^***^	0.12^**^	0.34^***^	− 0.053	0.040	− 0.024
Poor health (1)		1.81^***^		1.87***		1.89^***^
Fuel poor (1)		0.31^***^		0.79^***^		0.042
Age	0.0078^***^	0.0094^***^	0.0071^***^	0.0089^***^	0.0067^***^	0.0092^***^
Medium education	− 0.053^**^	− 0.0032	− 0.064^***^	− 0.067	− 0.048^**^	− 0.023
High education	− 0.16^***^	− 0.20^***^	− 0.19^***^	− 0.29^***^	− 0.19^***^	− 0.18^***^
Married/cohabiting	− 0.056^**^	− 0.032	− 0.052^**^	− 0.025	− 0.084^***^	− 0.048
Household resp. employed	− 0.16^***^	− 0.051	− 0.15^***^	− 0.052	− 0.16^***^	− 0.071^*^
# dependent children	− 0.012	0.059^**^	− 0.0062	0.062^**^	− 0.0084	0.049^**^
Hhold income (log)	− 0.10^***^	− 0.044	− 0.11^***^	− 0.043	− 0.12^***^	− 0.041
Constant	− 0.47^**^	0.25	− 0.34^*^	0.90	− 0.21	1.63^***^
*Energy poverty*						
Poor health (*t* − 1)	0.30^***^	0.10^**^	0.30^***^	− 0.069	0.081^***^	0.0062
Fuel poor (*t *− 1)	1.75^***^	0.81^***^	2.02^***^	0.90^***^	1.04^***^	0.60^***^
Poor health (1)	− 0.012^***^	0.30^***^	− 0.007^***^	0.48^***^		0.094^*^
Fuel poor (1)	0.033	1.38^***^	0.087^**^	1.90^***^		0.55^***^
Age	− 0.17^***^	− 0.018^***^	− 0.094	− 0.0093^***^	− 0.0012	− 0.0027^*^
Medium education	− 0.14^***^	0.057	− 0.40^***^	0.085	− 0.025	− 0.0085
High education	0.044	− 0.22^***^	− 0.12^**^	− 0.080	− 0.14^***^	− 0.098^*^
Married/cohabiting	0.068^***^	− 0.13^**^	0.031	− 0.42^***^	0.32^***^	0.087
Household resp. employed	− 0.36^***^	0.0034	− 0.32^***^	− 0.14	− 0.15^***^	− 0.15^***^
# dependent children	− 0.012^***^	0.060^**^	− 0.0071^***^	− 0.065	0.14^***^	0.19^***^
Hhold income (log)	0.033	− 0.27^***^	0.087^**^	− 0.17^***^	− 1.03^***^	− 1.08^***^
Constant	2.54^***^	5.75^***^	1.68^***^	8.37^***^	8.90^***^	12.9^***^
Year dummies	Yes	Yes	Yes	Yes	Yes	Yes
Regional dummies	Yes	Yes	Yes	Yes	Yes	Yes
Longitudinal average vars	No	Yes	No	Yes	No	Yes
Ρ		0.10^***^		0.024		0.039
${\sigma }_{\alpha 1}$		0.82^***^		0.99^***^		0.56^***^
${\sigma }_{\alpha 2}$		1.07^***^		1.04^***^		1.07^***^
${\rho }_{\alpha }$		0.18^***^		0.28^***^		0.088^*^
*Log-likelihood*	− 18,372.4	− 16,237.5	− 5194.3	− 5257.6	− 21,784.7	− 19,670.5
*Observations*	38,038	38,038	37,477	37,477	48,510	48,510

I Univariate pooled probit

II Bivariate dynamic probit with Wooldridge method

^*^p < 0.10, ^**^p < 0.05, ^***^p < 0.01

**Table 13:** Dynamic bivariate pooled probit model: health measured by mental health using SF-36 and all measures of energy poverty

	Nopaybills		Noheat^*^		Linchcost	
	I	II	I	II	I	II
*Poor health*						
Poor health (*t* − 1)	1.56^***^	0.65^***^	1.57^***^		1.60^***^	0.61^***^
Fuel poor (*t* − 1)	0.29^***^	0.061	0.37^***^		0.076^**^	0.019
Poor health (1)		1.21^***^				1.36^***^
Fuel poor (1)		0.25^***^				0.12^*^
Age	− 0.0066^***^	− 0.011^***^	− 0.007^***^		− 0.008^***^	− 0.013^***^
Medium education	− 0.087^***^	− 0.084^*^	− 0.094^***^		− 0.067^***^	− 0.0072
High education	− 0.032	0.027	− 0.051		− 0.071^***^	0.051
Married/cohabiting	− 0.13^***^	− 0.19^***^	− 0.124^***^		− 0.14^***^	− 0.18^***^
Household resp. employed	− 0.17^***^	− 0.10^**^	− 0.155^***^		− 0.19^***^	− 0.13^***^
# dependent children	0.00052	0.060^***^	0.008		0.0013	0.054^***^
Hhold income (log)	− 0.11^***^	0.021	− 0.119^***^		− 0.12^***^	0.0035
Constant	0.17	2.73^***^	0.279		0.305^*^	2.71^***^
*Energy poverty*						
Poor health (*t* − 1)	0.29^***^	0.092^*^	0.35^***^		0.022	− 0.018
Fuel poor (*t *− 1)	1.74^***^	0.83^***^	1.95^***^		1.04^***^	0.60^***^
Poor health (1)		0.18^***^				− 0.070
Fuel poor (1)		1.33^***^				0.50^***^
Age	− 0.010^***^	− 0.016^***^	0.005^***^		− 0.0012	− 0.0037^**^
Medium education	0.035	0.054	0.055		− 0.031	− 0.017
High education	− 0.17^***^	− 0.12^*^	− 0.169^***^		− 0.14^***^	− 0.090^*^
Married/cohabiting	− 0.11^***^	− 0.084	− 0.359^***^		0.31^***^	0.10^*^
Household resp. employed	0.055^*^	0.034	− 0.159^***^		− 0.17^***^	− 0.17^***^
# dependent children	0.061^***^	0.061^***^	0.031		0.14^***^	0.20^***^
Hhold income (log)	− 0.36^***^	− 0.25^***^	− 0.320^***^		− 1.02^***^	− 1.09^***^
Constant	2.47^***^	6.97^***^	1.616^***^		8.92^***^	13.5^***^
Year dummies	Yes	Yes	Yes		Yes	Yes
Regional dummies	Yes	Yes	Yes		Yes	Yes
Longitudinal average vars	No	Yes	No		No	Yes
Ρ		0.077^***^				0.0076
${\sigma }_{\alpha 1}$		0.81^***^				0.57^***^
${\sigma }_{\alpha 2}$		0.87^***^				0.92^***^
${\rho }_{\alpha }$		0.31^***^				0.086^**^
*Log-likelihood*	− 18,671.5	− 16,823.1	− 12,958.4		− 22,047.0	− 20,252.8
*Observations*	41,173	41,173	40,524		52,866	52,866

I Univariate pooled probit

II Bivariate dynamic probit with Wooldridge method

^*^p < 0.10, ^**^p < 0.05, ^***^p < 0.01

^Results did not converge for specification II

Results provide a mixed picture of potential cross-dependency between poor health and energy poverty. Across all three measures of health, if we do not control for initial conditions there is statistically significant evidence of cross-dependency effects. However, once we control for initial conditions the magnitude of the lagged coefficients is significantly reduced and, in many cases, no longer significant. In column II, in Tables 11, 12, and 13, we find no evidence of cross-dependency for the objective measures of energy poverty (linchcost). However, for our subjective measures of energy poverty we find some evidence of cross-dependency effects for being behind in bill payments when health is measured using general health from the SF-36 (Table 11, column 2) and with self-assessed health (Table 13 column 2).

When looking at the other coefficients included in the model, the results are consistent with our hypotheses. Higher income is significantly associated with a decreased likelihood of being in poor health and energy poverty. Married/cohabiting individuals are less likely to report poor health. The results are consistent across the different ways of measuring health and energy poverty and are similar to those found in the univariate probit models (Tables 5, 6, 7, 8, 9 and 10).

## Discussion and conclusion

This paper makes an important contribution to the literature by providing evidence on cross-dependency effects of health and energy poverty. This builds on existing causal research (Churchill and Smyth (2020) and Kahouli (2020)) by exploring the bi-directionality of health and energy poverty as well as earlier research estimating associations between energy poverty and health (Oliveras et al. 2020; Rodríguez-Álvarez et al. 2019; Thomson et al. 2017; Azpitarte et al. 2015; Lacroix and Chaton 2015; Thomson and Snell 2013).

We find mixed evidence for cross-dependency effects between energy poverty and poor health which are dependent upon how both energy poverty and health are measured. We find stronger evidence of cross-dependency effects for subjective measures of energy poverty whereas we do not find cross-dependency effects with health and objective measures of energy poverty (low income/high cost). If people feel like they are in energy poverty this can create a dynamically inter-related process or a ‘trap’ that needs to be accounted for in both energy and health policy to reduce health inequalities and help the most vulnerable in society. Controlling for initial conditions compared to not explicitly modelling the first observation of health and energy poverty in the results does have an impact on the magnitude and in some cases significance of the lagged coefficients on health and energy poverty and on the evidence of statistically significant cross-dependency effects.

This adds to the growing literature showing the inter-relatedness of poverty and its determinants/outcomes (Sweet et al. 2013; Clayton et al. 2015; Devicienti and Poggi 2011; Ribar and Hamrick 2003). Going forward researchers, should consider how using subjective measures may impact on estimating the causal relationships between poverty and the outcomes of poverty should consider the inter-relatedness of poverty and subjective determinants/outcomes to reduce the risk of bias.

Similar to the literature estimating a causal relationship between energy poverty and health (Churchill and Smyth (2020) and Kahouli (2020)) we find a significant relationship in our univariate models between poor health and energy poverty which ranges between 0.10 and 0.35 which is very similar to the findings from Churchill and Smyth (2020) where they found a relationship of energy poverty on poor health ranging from 0.10 to 0.30. What we add to the literature is greater understanding of the mechanisms behind this causal relationship specifically around the potential impact of traps or of cross-dependency effects between subjective energy poverty and health.

In regards to policy, our findings highlight that for subjective energy poverty or feelings of financial stress and debt that this may have an inter-related dynamic relationship with health and in particular mental health. As the identification of potentially vulnerable groups is often difficult in practice, the existence of these spillover effects may be of use for policy makers committed to combat disadvantage and overall citizens’ wellbeing. For subjective energy poverty in particular, debt advice, energy savings tips, and advice on how to keep warm without relying on energy consumption to those struggling at other venues where individuals seek support for other financial difficulties such as food banks may be a cost-effective way to reduce energy poverty and improve health and identify at risk groups.

In the study we used a balanced panel of respondents who respond in all waves. This may impact on the generalisability of the findings. Our results may be a lower bound estimate if those who are more likely to be in poor health and energy poverty exit the sample. However, by controlling for initial conditions which controls for individual characteristics at baseline this reduces some of the biased introduced from a balanced panel. It is likely that income may be endogenously related to both health and energy poverty. Thus, we would not want to make strong recommendations on how increasing income would impact on energy poverty or health or the relationship between the two given the uncertainty around the true magnitude and significance of the coefficient. Especially as our results suggest that subjective financial status matters and thus increasing income depending upon the level of the increase may not necessarily relieve feelings of subjective financial hardship.

In addition, we do not have information on housing quality. Living in poor quality housing is associated with increased risk of energy poverty. Many low income families cannot afford to make their homes more energy efficient. If households are renting, many private landlords do not invest in energy efficiency because of the costs and administrative burden (Jessel et al. 2019). Additional people living in manufactured housing just as mobile, or trailer homes are severely impacted by physical energy insecurity because of poor insulation and weather optimization of these types of homes (Jessel et al. 2019). Thus, poor quality housing is likely to be an unobserved factor in our analysis which may explain both poor health and energy poverty potentially inflating our estimated coefficients of past health and past energy poverty on current outcomes. We also do not look at severity of health which may have some impact on cross-dependency effects between health and energy poverty which should be explored in future research.

This paper makes an important first step in understanding if and how energy poverty and health are inter-related over time. Going forward the climate emergency is going to make energy poverty increasingly important global issue and necessary to address to reduce health inequalities and improve overall health. This research highlights that there is some evidence for subjective measures of energy poverty of an inter-related relationship with health and in particular mental health.

## Data Availability

This paper uses unit record data from the Household, Income and Labour Dynamics in Australia (HILDA) Survey. The HILDA Project was initiated and is funded by the Australian Government Department of Social Services (DSS) and is managed by the Melbourne Institute of Applied Economic and Social Research (Melbourne Institute). The findings and views reported in this paper, however, are those of the author and should not be attributed to either DSS or the Melbourne Institute. All data are available to download for users who register with Australian Data Archive. More information can be found at: https://dataverse.ada.edu.au/dataverse/hilda.

## Footnotes

1 https://ec.europa.eu/energy/content/possible-approach-define-energy-poverty-inability-keep-home-adequately-warm_en. Accessed 18 September 2020.

2 https://assets.publishing.service.gov.uk/government/uploads/system/uploads/attachment_data/file/882404/annual-fuel-poverty-statistics-report-2020-2018-data.pdf. Accessed 18 September 2020.

3 Fuel poverty and energy poverty are used interchangeably in this paper.

4 https://assets.kpmg/content/dam/kpmg/au/pdf/2017/census-insights-energy-poverty-australia.pdf. Accessed 18 September 2020.

5 https://www.gov.uk/government/publications/fuel-poverty-a-framework-for-future-action.

6 Since Wave 5 (2005) of HILDA Survey, respondents have been asked about household expenditure on electricity, gas and other heating fuel (e.g., firewood and heating oil).

7 Moreover, this approach results in substantial savings in computer time, particularly relevant in cases like ours with more than 38,000 (general health) and 41,000 observations (mental health) per regression estimated.

## Code availability

All code used in the analysis is available on request to the authors.
